# Functional Recovery Predictors in Hip Fractures: Insights from a Community Clinical Pathway

**DOI:** 10.3390/jcm14103430

**Published:** 2025-05-14

**Authors:** Ai Takahashi, Hiroaki Naruse, Daiki Hasegawa, Hideaki Nakajima, Akihiko Matsumine

**Affiliations:** 1 Department of Orthopaedics and Rehabilitation Medicine, University of Fukui, 23-3 Matsuoka Shimoaizuki, Eiheiji-cho, Yoshida-gun, Fukui 910-1193, Japan; aitun@u-fukui.ac.jp (A.T.); matsumin@u-fukui.ac.jp (A.M.); 2 Division of Rehabilitation Medicine, University of Fukui Hospital, 23-3 Matsuoka Shimoaizuki, Eiheiji-cho, Yoshida-gun, Fukui 910-1193, Japan; narunia@u-fukui.ac.jp (H.N.); mdaiki@g.u-fukui.ac.jp (D.H.)

**Keywords:** hip fractures, orthopaedic nursing, rehabilitation, walking

## Abstract

**Background:** Osteoporotic hip fractures in the elderly significantly impact mobility and quality of life. Optimising early management is crucial for improving the functional outcomes. **Methods:** This single-centre retrospective cohort study included patients with femoral trochanteric (n = 142) or femoral neck fractures (n = 127) treated between January 2016 and March 2023. The patients were divided into ambulatory and non-ambulatory groups based on their walking ability at discharge from the rehabilitation hospital. The explanatory variables included age, sex, fracture type, surgical method, pre-surgical days, hospital days, dementia, and the Nursing Needs Score (NNS). **Results:** The most common age group was 85–89 years old. Only 23.4% of patients underwent surgery within 2 days of admission. The median hospital stay was 20 days in acute care and 52 days in rehabilitation hospitals. Walking ability declined in 66.9% of the patients. Pre-injury mobility and acute care hospital NNS were identified as independent predictors of ambulatory outcomes. **Conclusions:** Pre-injury mobility and the Nursing Needs Score (NNS) assessed at the acute care hospital were identified as critical determinants of postoperative ambulatory status. These findings highlight the importance of community collaboration and preventive rehabilitation strategies aimed at improving basic mobility, maintaining cognitive function, and preserving walking ability.

## 1. Introduction

Hip fractures are one of the most serious fragility fractures caused by osteoporosis in the elderly. Hip fractures are classified into femoral neck and femoral trochanteric fractures, which increase mortality rates and lead to a need for nursing care due to reduced mobility. Hip fractures are a medical and social problem specific to the ageing society, leading to complications such as pneumonia and dementia after the fracture. The Japanese Orthopaedic Association Database (JOANAR) is the largest orthopaedic database in Japan, with over 2300 registered facilities. According to the 2023 JOANAR Annual Report, the surgical treatment of proximal femoral fractures constitutes the largest proportion of orthopaedic procedures in Japan. A total of 1,076,211 patients were analysed, of whom 113,033 (10.5%) underwent osteosynthesis for proximal femoral fractures and 70,835 (6.6%) underwent total hip replacement. Surgery for proximal femoral fractures accounted for 17.1% of the total [1]. If we include cases where surgery was not performed due to complications or other reasons, the number of potential patients is expected to increase. In a previous survey of hip fractures, 15% of patients received conservative treatment [2], while according to a Japanese long-term care insurance system survey, 13.9% of cases requiring nursing care are due to fractures or falls [3]. Hence, appropriate management of hip fractures is important from both medical and economic perspectives.

Community medical pathways have been used for hip fractures to maximise treatment efficacy. The clinical pathway is a treatment plan shared by acute care, rehabilitation, and family doctors, and includes specific details regarding the treatment content, progress, and final goals of each facility. In addition, all affiliated hospitals use the same format. A meeting regarding the clinical pathway is held every four months, where doctors, nurses, rehabilitation staff, medical social workers, and administrative staff from the associated hospitals discuss the operational status. In recent years, the Fracture Liaison Service (FLS) has demonstrated the utility of multidisciplinary assessment and treatment in patients with osteoporotic fractures. FLS consists of patient pick-up, assessment of fracture risk, initiation and continuation of treatment, and follow-up to prevent secondary fractures in patients who have experienced fragility fractures. In Japan, the Fragility Fracture Network Japan (FFN-Japan) is the parent organisation for the development of a database and optimisation of treatment, mainly for hip fracture patients [4]. FLS teams have been set up in each hospital to work in collaboration with FFN-Japan. FLS is a continuous patient service that does not end at the acute hospital alone but needs to be continued to the rehabilitation hospital and the family doctor. Therefore, the clinical pathway of hip fractures is useful in FLS. The clinical pathway meeting functions as a regional version of the FLS.

This study aimed to utilise regional medical pathway data to ascertain the actual state of femoral neck fracture treatment and identify future issues. The primary objective was to identify factors influencing functional outcomes after hip fracture surgery, while the secondary objective was to quantify the acute-phase management that contributed to improved patient performance.

## 2. Materials and Methods

### 2.1. Study Population and Assessment Items

**Figure 1:**
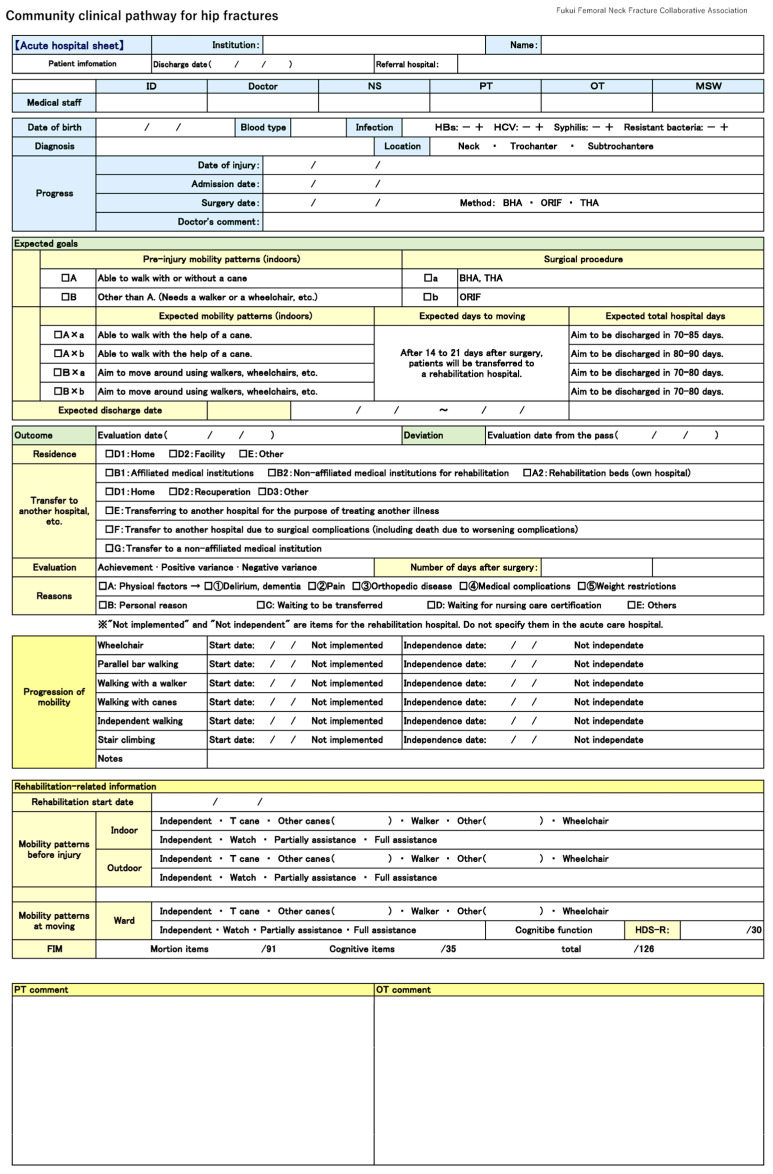

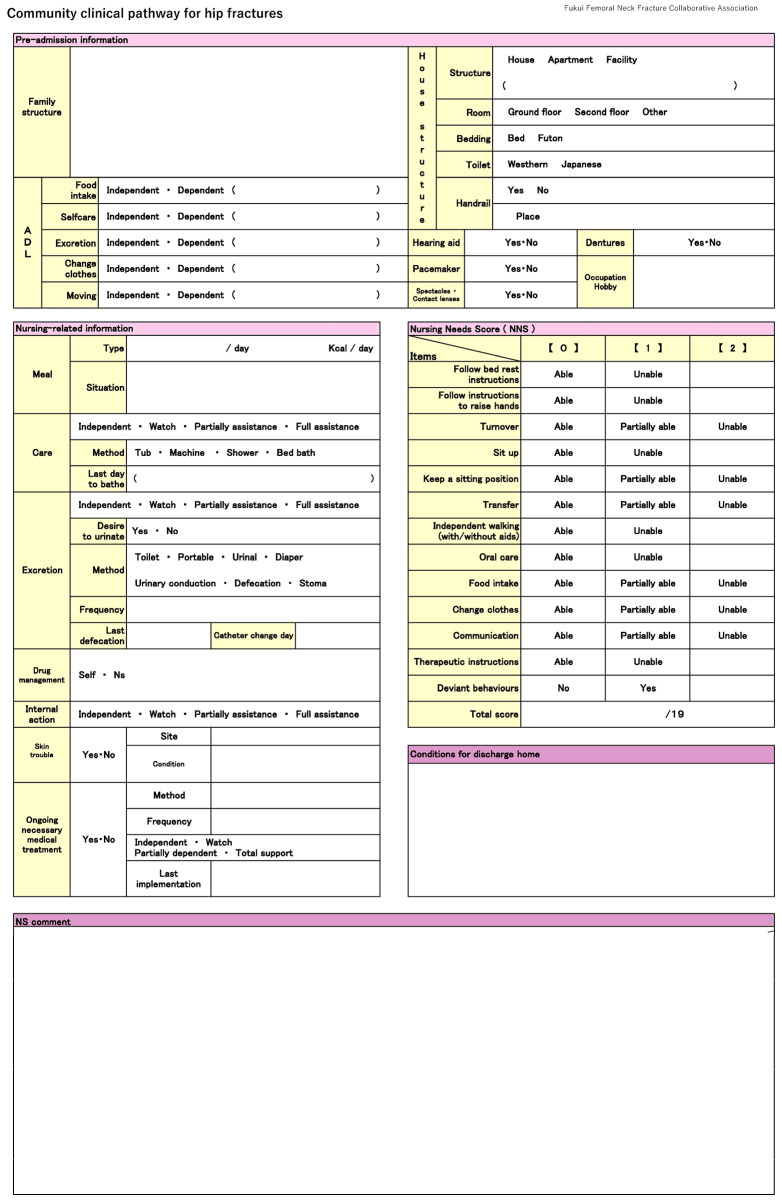

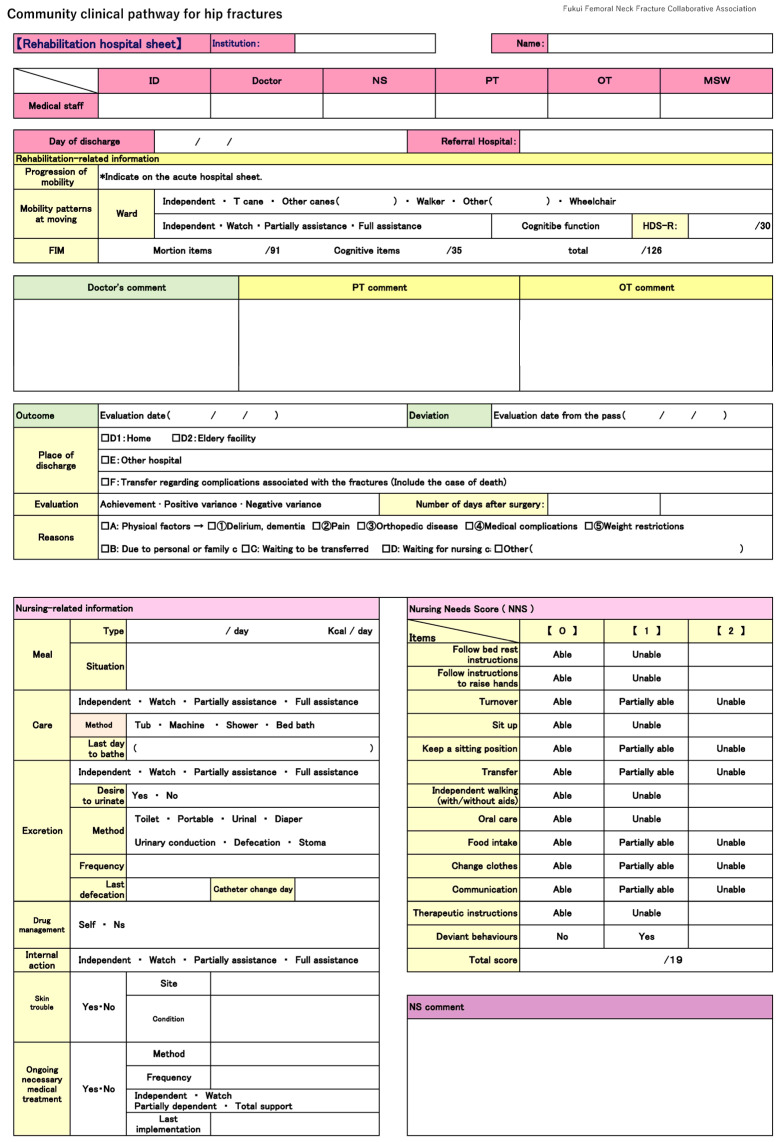

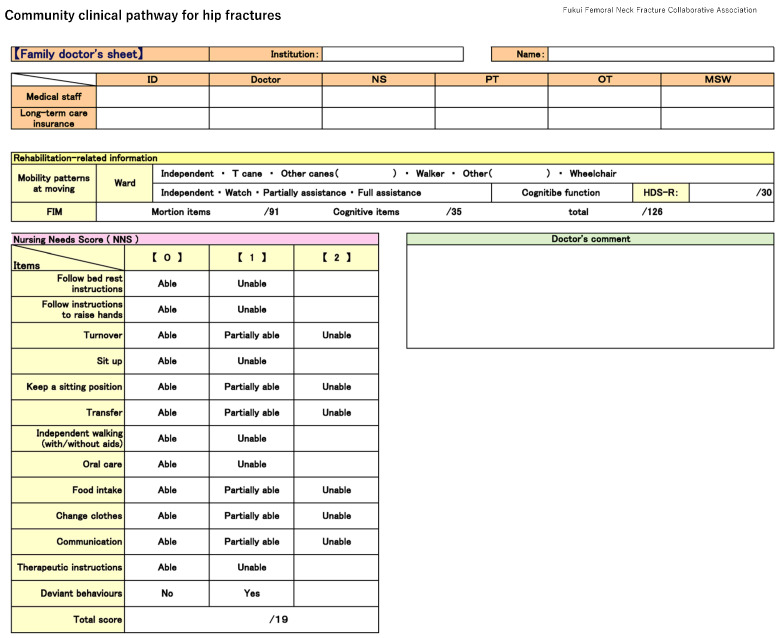
Community clinical pathways for hip fractures.

This single-centre retrospective cohort study included 346 patients who were admitted to the University of Fukui Hospital with a diagnosis of femoral trochanteric (AO/OTA 31-A) or femoral neck fracture (31-B) between January 2016 and March 2023 and underwent surgical treatment. The patients were identified using the ICD-10 codes S72.00 and S72.10. Fracture type was confirmed by board-certified orthopaedic surgeons using plain radiographs or CT scans and classified according to the AO/Orthopaedic Trauma Association (AO/OTA) system [5]. Patients with high-energy trauma (e.g., traffic accidents, falls from height), pathological fractures due to bone tumours, or metabolic bone diseases other than osteoporosis were excluded from this study. Only fragility fractures resulting from low-energy mechanisms were included in this study. The community clinical pathway for hip fractures (Figure 1) was used to coordinate care between acute care and rehabilitation hospitals. Data were extracted from this pathway and analysed anonymously. The assessment items included patient age, sex, pre-injury mobility, fracture type, surgical method, days from admission to surgery, total hospital stay, presence of dementia, and the Nursing Needs Score (NNS). The NNS is a Japanese index used to assess patient dependency and required nursing care. The number of days related to treatment timelines was also calculated from the pathway data. The primary outcome was the ambulatory status at discharge from the rehabilitation hospital. The explanatory variables included all the assessment items listed above. Surgical procedures comprised osteosynthesis techniques (e.g., gamma nail, dynamic hip screw [DHS], and Hansson pin) and bipolar head arthroplasty (BHA). Surgical methods were determined through interdisciplinary case conferences in each hospital based on the patient’s condition, fracture characteristics, and institutional protocols. Treatment approaches during the rehabilitation phase varied by institution but typically included physical and occupational therapy focused on basic mobility, activities of daily living (ADL), muscle strengthening, range of motion, and gait training. The evaluation items within the clinical pathway were completed collaboratively by the medical staff and shared in a standardised format across institutions. After rehabilitation, the completed data were returned to the originating acute care hospital, and aggregation was managed by medical social workers. This study was approved by the Ethics Committee of the University of Fukui (approval number: 20240159).

### 2.2. Clinical Assessment

The clinical pathway included the patient’s age, sex, blood group, fracture type, date of surgery, surgical procedure, pre-injury ambulatory ability, place of residence, and the discharge institution. In this pathway, the expected mobility outcome and corresponding hospital stay were predefined based on the patient’s pre-injury walking ability and type of surgery. For example, patients who were independently ambulatory prior to injury and underwent bipolar head arthroplasty (BHA) were expected to regain independent or assisted walking within a hospital stay of 70–85 days. Similarly, if a patient walked independently before the injury and underwent osteosynthesis, the length of stay in the rehabilitation hospital was extended to a maximum of 90 days. These expectations served as benchmarks for evaluating progress and outcomes within the pathways.

Rehabilitation details included the date of the first postoperative wheelchair ride, the date at the start of standing on the parallel bars, the date at the start of walking with a walker, the date at the start of walking with a cane, and whether each movement was independent. Mobility patterns were assessed before the injury and upon discharge from the acute and rehabilitation hospitals, which were classified into the following categories: independent, with walking aids (T-cane, other canes, and walkers), and wheelchair use. Those who were categorised as ‘wheelchair users’ at the time of discharge from the rehabilitation hospital were defined as the non-ambulatory group, while those who were categorised as ‘independent’ and ‘with walking aids’ were defined as the ambulatory group. ADL at discharge were assessed separately for motor and cognitive items using the Functional Independence Measure (FIM).

Nursing aspects included family structure, housing arrangements, pre-injury basic movement skills, medication, and the Nursing Needs Score (NNS). The NNS is a scoring system that assesses nursing effort (Table 1) and rates the ability of the patient to perform basic activities on a scale of 0–19, with higher scores indicating greater nursing needs. The NNS includes the following assessments: follow bed rest instructions, follow instructions to raise hands, turnover, sit up, keep a sitting position, transfer, independent walking (with/without aids), oral care, food intake, change clothes, communication, therapeutic instructions, and deviant behaviours. The NNS is widely used in acute care hospitals across Japan and has been incorporated into facility standards under the National Health Insurance System.

### 2.3. Statistical Analysis

A full-case analysis was performed by excluding samples with missing values during data preprocessing. Outliers, defined as values greater than three standard deviations from the mean, were also excluded. Data from 269 patients were analysed (142 with trochanteric fractures and 127 with femoral neck fractures). Multivariate analysis was conducted using complete case analysis, and cases with missing data for the explanatory variables were excluded from the model. Univariate analysis was performed to examine the relationship between clinical characteristics and ambulatory status at discharge. Normality tests were performed for continuous variables. For normally distributed variables (e.g., length of hospital stay), *t*-tests were applied, and for non-normally distributed variables (e.g., age, pre-surgical days, and the NNS), the Mann–Whitney U test was used. Categorical variables, such as sex, fracture type, surgical method, and dementia status, were compared using the chi-squared test. To identify patient and hospital factors affecting postoperative ambulation, multiple logistic regression analysis was performed using age, pre-injury mobility, pre-surgical days, acute care hospital NNS score, and discharge dementia status as independent variables. The items assessed in the multivariate analysis were derived from the results of the univariate analysis, optimal number of items for the sample size, and previous reports. Spearman’s correlation analysis was used to investigate associations between items in the multivariate analysis. Statistical significance was set at *p* < 0.05. All statistical analyses were performed using the R-4.4.1.

## 3. Results

### 3.1. Patient Characteristics and Timing of Surgery

**Figure 2:**
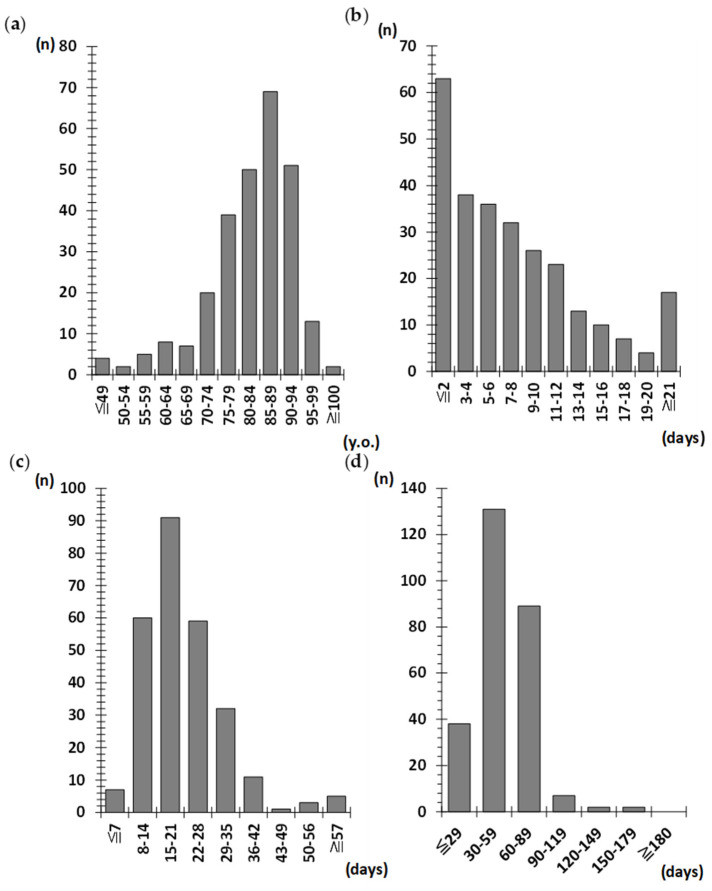
Histograms of (**a**) patient age, (**b**) pre-surgical days, (**c**) hospital days in an acute care hospital, and (**d**) rehabilitation hospital. Patient age showed a unimodal pattern, with a peak of 85–89 years (**a**). In total, 23.4% and 50.9% of patients underwent surgery within two and seven days of admission, respectively (**b**). The duration of stay in acute-care hospitals was 15–21 days in the largest proportion (**c**). The most common length of stay in rehabilitation hospitals was 30–59 days. Overall, 96.0% of the patients were discharged from the rehabilitation hospital within 90 days (**d**).

Patient characteristics are presented in Table 2 and Figure 2. Patient age was non-normally distributed, with a peak between 85 and 89 years. Of the 269 patients, 67.7% were aged ≥80 years (Figure 2a). Of these patients, 73.2% were female. The pre-injury mobility pattern was 67.7% independent, 27.5% walking aid users, and 4.8% wheelchair users; 95.2% of all the patients could walk. Only 23.4% of the patients underwent surgery within 2 days of admission, and the median number of pre-surgical days was 6 days (Figure 2b). A total of 33.8% of the patients were discharged from acute care hospitals within 15–21 days (Figure 2c), and 45.0% were discharged from rehabilitation hospitals within 61–90 days (Figure 2d).

### 3.2. Changes in Walking Ability

**Figure 3:**
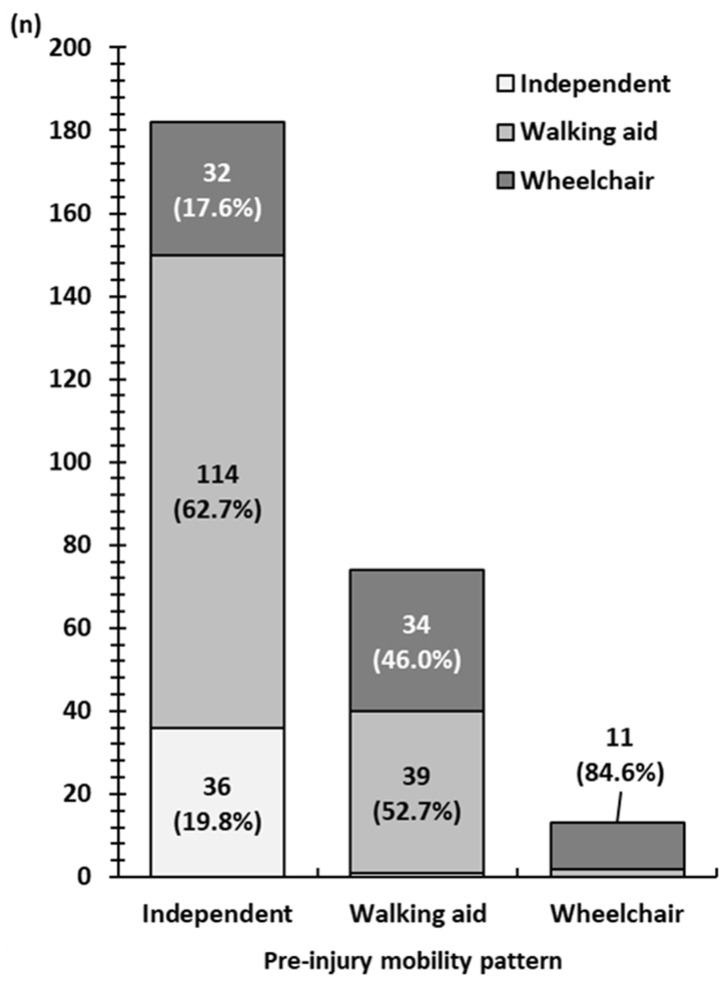
Pre-injury mobility pattern and corresponding walking ability at discharge from the rehabilitation hospital. Of the patients with an independent pre-injury mobility pattern, 80.3% required a walking aid or wheelchair.

Walking ability at discharge from the rehabilitation hospital for each pre-injury mobility pattern is shown in Figure 3. The horizontal axis of the graph shows the pre-injury mobility pattern (independent, with walking aids, and wheelchair users), and the vertical axis shows the number of people corresponding to each mobility pattern at discharge from rehabilitation hospitals. Regarding outcomes, 19.8% of the patients whose pre-injury mobility pattern was independent walking maintained independent ambulation, 62.7% became walking aid users, and 17.6% became wheelchair users; 46.0% of patients whose pre-injury mobility pattern was using a walking aid became wheelchair users. Walking ability decreased in 66.9% of all patients.

### 3.3. Factors Influencing Mobility

Factors affecting mobility at discharge from rehabilitation hospitals were investigated. The univariate analysis showed positive correlations for pre-injury mobility and acute care hospital discharge mobility and negative correlations for age, pre-injury dementia, acute and rehabilitation hospital NNS, and acute and rehabilitation hospital discharge dementia. However, no significant differences were found in the fracture type, surgical procedure, preoperative days, or length of hospital stay (Table 3). The logistic regression analysis of pre-injury and acute phase factors revealed that preoperative mobility and acute care hospital NNS had a significant effect on the dependent variable. Patient age tended to influence mobility during rehabilitative hospital discharge (Table 4).

## 4. Discussion

Osteoporotic fractures are among the most significant medical and social problems. Osteoporosis is mainly caused by post-menopausal oestrogen deficiency, resulting in fragility fractures such as distal radius, proximal humerus, vertebral compression, and hip fractures. Among these osteoporotic fractures, hip fractures are directly related to ADL decline and mortality, and the prevention and recurrence of fractures are important. The most common causes of long-term care are dementia (16.6%), cerebrovascular disease (16.1%), and fractures/falls (13.9%), all of which require long-term care insurance [3]. A nationwide survey conducted by Orimo et al. [6] estimated that 37,600 men and 138,100 women sustained hip fractures in 2012 (175,700 patients). According to the JOANAR, the Japanese Orthopaedic Association database, hip fracture surgeries exceeded 170,000 cases in 2023, representing the largest number of orthopaedic surgeries [1]. However, recent epidemiological studies have reported a decrease in the incidence of hip fractures in many countries [7,8,9]. A recent survey in Japan indicated that the increase in the incidence of hip fractures has slowed [10]. On the other hand, Japan has the highest proportion of elderly people worldwide [11], and hip fractures are expected to remain a significant health issue for the elderly for several decades. Factors affecting functional recovery after hip fractures include (1) medical factors, such as the presence of comorbidities; (2) surgical factors, such as delayed surgery; (3) socioeconomic factors, such as age, sex, and ethnicity; and (4) systemic factors, such as centres with low caseloads [12]. Most patients are transferred to acute care hospitals, where the following risks are assessed and corrected for early surgical treatment: traumatic anaemia, respiratory and cardiovascular diseases such as chronic obstructive pulmonary disease and severe arrhythmias, renal diseases, and endocrine disorders such as diabetes mellitus. After treatment in an acute care hospital, the patients are transferred to a rehabilitation hospital. The final discharge destination is the home or nursing home, where care is continued by the home physician. Clinical pathways are used to provide appropriate hip fracture treatment and continuity of care from the acute phase to the recovery phase and into the maintenance phase. In this study, factors predicting the functional outcomes of hip fractures were identified based on data from the community medical pathway for hip fractures.

Although previous reports have shown that advanced age is a factor affecting functional prognosis after hip fractures [13,14,15], age was not a statistically significant predictor in our logistic regression model (*p* = 0.06). This discrepancy may reflect confounding by other variables, such as pre-injury mobility or nursing care needs. The proportion of the Japanese population aged ≥65 years has continued to increase. In 2023, 29.1% of the total population was aged ≥65 years, and it is predicted that by 2070, 38.7% of the population will be ≥65 years [16]. Recent advances in surgical materials and medical technology have made surgery more accessible to high-risk patients who were previously treated conservatively. Decline in mobility in elderly patients is associated with musculoskeletal, respiratory, cardiovascular, and neurological functions. The usefulness of a multidisciplinary approach in ensuring successful rehabilitation after hip fractures has been reported [17,18,19]. Perspectives from geriatric and rehabilitation medicine are as important as orthopaedic treatment for functional recovery after hip fractures. This is a concept similar to FLS, which aims to improve ADL and prevent secondary fractures by providing thorough aftercare for patients with fragility fractures.

The impact of pre-injury mobility patterns on postoperative functional outcomes has been investigated in numerous recent studies. A previous study demonstrated that pre-injury gait function, assessed by the Functional Ambulation Category (FAC) score, exhibited a positive correlation with the FAC score at 6 months post-injury [2]. The results of the logistic regression analysis in the current study showed that pre-injury mobility patterns were identified as a factor affecting the ability to walk after discharge from rehabilitation hospitals, which supports previous studies [20,21,22]. This finding highlights the importance of maintaining mobility among healthy older adults and suggests that preventive rehabilitation approaches may help patients walk after hip fractures.

The National Institute for Health and Care Excellence guidelines stipulate the optimal timing of surgery for hip fractures, which was reduced from 48 h to 36 h in the 2023 revision [23]. Surgery on the same day or the day after admission is recommended, and the effectiveness of this approach has been demonstrated in terms of survival, complications, and economic costs. In this study, we included pre-surgical days as an explanatory variable in the multivariate regression analysis to evaluate its association with the functional outcomes. However, no significant associations were observed. Although surgical delay is often considered a confounding factor for functional recovery, our findings did not support this in the current cohort. This may be due to variations in patient risk profiles and preoperative optimisation requirements. Patients with minimal or no complications may have undergone early surgery, which could have been influenced by confounding factors. A Japanese study conducted between 2010 and 2014 revealed that only 22.5% of patients underwent surgery within 2 days of admission [24]. In the current survey, 23.4% of patients underwent surgery on the second day after admission. As the University of Fukui Hospital is the only advanced care hospital in Fukui Prefecture, many high-risk patients with serious complications, including hip fractures, are no exception. Perioperative risk factor assessment and treatment may require more days than recommended. In 2022, a revision of the medical payment system led to the introduction of additional compensation for early surgeries in Japan. A further reduction in the number of days spent in the pre-surgical period is anticipated in the coming years, which will also lead to enhanced functional recovery.

The NNS is an assessment tool introduced in 2008 to ensure the provision of adequate nursing services in accordance with the severity of the patient’s condition. This scoring system is commonly used in Japanese hospitals and consists of three items (A, B, and C). Item B was included in the clinical pathway, and the score was calculated on a 19-point scale, with higher scores indicating a greater need for assistance. NNSs have been reported to correlate with the incidence of pressure ulcers in acute care units [25]. Among the NNS items, basic actions such as ‘Turnover’ and ‘Transfer’ and items related to cognitive functions such as ‘Change clothes’ and ‘Communication’ have high scores. However, independent walking is not as important in the NNS assessment. This finding suggests that acute rehabilitation strategies are more multifaceted in the postoperative functional recovery of patients with hip fractures. In Japanese hospitals, the NNS is evaluated daily, except on the day of discharge, which is a shorter period than other ADL assessment scores, thereby reflecting the most recent condition of the patient. In this study, the NNS in an acute care hospital was identified as a predictor of functional prognosis, which has not been previously reported.

Previous studies demonstrated that dementia is a predictor of functional recovery [26,27,28], and the degree of independence in daily living among elderly people with dementia has been incorporated into clinical pathways. This assessment method is common in Japan and classifies the activities of older adults with dementia as grades I–IV and M [29]. This method makes judgments based on interview content; therefore, the results may vary depending on the understanding of the researcher and their experience with dementia. The current survey did not subdivide each grade, defining independent activity (grade I) as having no dementia and the other cases as having dementia. Patients with dementia are at high risk of falls due to decreased mobility caused by impaired balance and other factors. In addition, decreased attention and comprehension inhibit the rehabilitation of patients with dementia [30,31]. Dementia has also been associated with poor nutrition and osteoporosis, indicating a higher risk of fractures [32]. In the present study, dementia was not identified as a significant factor in the logistic regression analysis, which could be due to the significant correlation between dementia and the acute care hospital NNS (Supplementary Data).

It should be noted that this study had several limitations. First, the clinical pathway was not applied to all patients with hip fractures. This study only included patients who had undergone surgery, and some rehabilitation hospitals did not adopt the clinical pathway. Clinical paths were not implemented in hospitals where the number of hip-fracture patients was low or where clinical paths were deemed unnecessary for management reasons; hence, the exact population of patients with hip fractures is not known. Second, the data utilised in the present study were solely those returned to acute care hospitals from rehabilitation hospitals and various clinics. Selection bias may have influenced the results, given that the data collection rates varied between hospitals. Third, we did not verify the detailed medical histories of the patients, including osteoporosis status, cause of fracture, and treatment for osteoporosis. These limitations should be addressed in future analyses that use data from the Diagnosis Procedure Combination Study.

## 5. Conclusions

This retrospective cohort study using a community clinical pathway identified pre-injury mobility and the Nursing Needs Score (NNS) in acute care hospitals as key predictors of functional outcomes after hip fracture surgery. The NNS is one of the most frequently used assessment scores in Japanese nursing practice. The need for assistance in acute care hospitals has been shown to predict functional recovery, highlighting the importance of developing local rehabilitation and care plans with FLS teams that consider these factors.

## Figures and Tables

**Table 1:** Nursing Needs Score.

	Score		
Items	0	1	2
Follow bed rest instructions	Able	Unable	
Follow instructions to raise hands	Able	Unable	
Turnover	Able	Partially able	Able
Sit up	Able	Unable	
Keep a sitting position	Able	Partially able	Able
Transfer	Able	Partially able	Able
Independent walking (with/without aids)	Able	Unable	
Oral care	Able	Unable	
Food intake	Able	Partially able	Able
Change clothes	Able	Partially able	Able
Communication	Able	Partially able	Able
Therapeutic instructions	Able	Unable	
Deviant behaviours	No	Yes	
Total	19 points		

**Table 2:** Patient characteristics.

Variables		
Number of patients		269
Median age (range)		85 (43–101)
Female, n (%)		197 (73.2%)
Fracture type, n (%)		
	Trochanteric fracture (AO/OTA 31-A)	142 (52.7%)
	Neck fracture (AO/OTA 31-B)	127 (47.3%)
Pre-injury mobility pattern, n (%)		
	Independent	182 (67.7%)
	Walking aid	74 (27.5%)
	Wheelchair	13 (4.8%)
Surgical method, n (%)		
	Osteosynthesis	94 (34.9)
	Bipolar head arthroplasty	175 (65.1)
Median pre-surgical days (range)		6 (0–49)
Median hospital days (range)		
	Acute care hospital	20 (4–77)
	Rehabilitation hospitals	52 (5–154)
	Total hospitals	73 (20–186)
Pre-injury dementia, n (%)		147 (54.6)
Nursing Needs Score, median (range)		
	Acute care hospital	6 (0–18)
	Rehabilitation hospitals	1 (0–19)

**Table 3:** Group comparisons using univariate analysis.

Variable	Ambulatory Group (n = 192)	Non-Ambulatory Group (n = 77)	*p*-Value
Median age (range)	83 (43–101)	88 (71–100)	<0.01
Female, n (%)	145 (53.9%)	52 (19.3%)	0.24
Pre-injury mobility (wheelchair), n (%)	2 (1.0%)	11 (14.3%)	<0.01
Pre-injury dementia (dementia), n (%)	57 (29.7%)	65 (84.4%)	<0.01
Fracture type (neck), n (%)	96 (0.5%)	31 (40.3%)	0.21
Surgical method (BHA), n (%)	72 (37.5%)	22 (28.6%)	0.21
Pre-surgical days (range)	6 (0–49)	7 (0–49)	0.31
Acute care hospital days (range)	20 (4–67)	21 (7–77)	0.46
Rehabilitation hospital days (range)	51 (9–138)	56 (5–154)	0.58
Total hospital days (range)	72 (21–158)	79 (20–186)	0.49
Acute care hospital NNS (range)	5 (0–16)	11 (3–18)	<0.01
Rehabilitation hospital NNS (range)	0 (0–19)	6 (0–19)	<0.01
Acute care hospital discharge mobility (wheelchair), n (%)	144 (75.0%)	73 (94.8%)	<0.01
Acute care hospital discharge dementia, n (%)	111 (57.8%)	71 (92.2%)	<0.01
Rehabilitation hospital discharge dementia, n (%)	101 (52.6%)	73 (94.8%)	<0.01

BHA, bipolar head arthroplasty; NNS, Nursing Needs Score.

**Table 4:** Logistic regression analysis.

	Odds Ratio	95% Confidence Interval	*p*-Value
Age	0.95	0.90–1.00	0.06
Pre-injury mobility	2.99	1.67–5.34	<0.01
Pre-surgical days	1.01	0.97–1.05	0.54
Acute care hospital Nursing Needs Score	0.72	0.64–0.80	<0.01
Acute care hospital discharge dementia	1.84	0.62–5.51	0.27

## Data Availability

All data generated or used during this study are available from the corresponding author and first author upon reasonable request.

## Supplementary Materials

The following supporting information can be downloaded at: https://www.mdpi.com/article/10.3390/jcm14103430/s1, Table S1: Correlation coefficients between the factors.

## Abbreviations

The following abbreviations are used in this manuscript:

ADL	Activities of daily living
BHA	Bipolar head arthroplasty
FAC	Functional Ambulation Category
FIM	Functional Independence Measure
FLS	Fracture Liaison Service
JOANAR	Japanese Orthopaedic Association Nation Registry
NNS	Nursing Needs Score

## References

[1] The Japanese Orthopaedic Association (2023). The Database of Japanese Orthopaedic Association National Registry (JOANR). 2023 Annual Report. https://www.joa.or.jp/joa/files/JOANR_annual_report_2023.pdf.

[2] Takahashi A., Naruse H., Kitade I., Shimada S., Tsubokawa M., Kokubo Y., Matsumine A. (2020). Functional outcomes after the treatment of hip fracture. PLoS ONE.

[3] Ministry of Health, Labour and Welfare, Japan (2022). Comprehensive Survey of Living Conditions 2022. https://www.mhlw.go.jp/toukei/saikin/hw/k-tyosa/k-tyosa22/dl/05.pdf.

[4] Fragility Fracture Network Japan (FFN-Japan) About FFN-Japan. https://ffn.or.jp/about_en/.

[5] (2018). AO/OTA Fracture and Dislocation Classification Compendium. https://classification.aoeducation.org/.

[6] Orimo H., Yaegashi Y., Hosoi T., Fukushima Y., Onoda T., Hashimoto T., Sakata K. (2016). Hip fracture incidence in Japan: Estimates of new patients in 2012 and 25-year trends. Osteoporos. Int..

[7] Michaëlsson K., Baron J.A., Byberg L., Larsson S.C., Melhus H., Gedeborg R. (2024). Declining hip fracture burden in Sweden 1998–2019 and consequences for projections through 2050. Sci. Rep..

[8] Swayambunathan J., Dasgupta A., Rosenberg P.S., Hannan M.T., Kiel D.P., Bhattacharyya T. (2020). Incidence of hip fracture over 4 decades in the Framingham heart study. JAMA Intern. Med..

[9] Remily E.A., Mohamed N.S., Wilkie W.A., Mahajan A.K., Patel N.G., Andrews T.J., Nace J., Delanois R.E. (2020). Hip fracture trends in America between 2009 and 2016. Geriatr. Orthop. Surg. Rehabil.

[10] Takusari E., Sakata K., Hashimoto T., Fukushima Y., Nakamura T., Orimo H. (2021). Trends in hip fracture incidence in Japan: Estimates based on nationwide hip fracture surveys from 1992 to 2017. JBMR. Plus.

[11] United Nations, Department of Economic and Social Affairs, Population Division (2019). World Population Ageing 2019: Highlights.

[12] Xu B.Y., Yan S., Low L.L., Vasanwala F.F., Low S.G. (2019). Predictors of poor functional outcomes and mortality in patients with hip fracture: A systematic review. B.M.C. Musculoskelet. Disord..

[13] Buecking B., Bohl K., Eschbach D., Bliemel C., Aigner R., Balzer-Geldsetzer M., Dodel R., Ruchholtz S., Debus F. (2015). Factors influencing the progress of mobilization in hip fracture patients during the early postsurgical period?—A prospective observational study. Arch. Gerontol. Geriatr..

[14] Kristensen M.T., Foss N.B., Kehlet H. (2009). Factors with independent influence on the ‘timed up and go’ test in patients with hip fracture. Physiother. Res. Int..

[15] Hulsbæk S., Larsen R.F., Troelsen A. (2015). Predictors of not regaining basic mobility after hip fracture surgery. Disabil. Rehabil..

[16] Cabinet Office, Government of Japan (2022). Annual Report on the Ageing Society: Summary FY2022. https://www8.cao.go.jp/kourei/english/annualreport/2022/pdf/2022.pdf.

[17] Prestmo A., Hagen G., Sletvold O., Helbostad J.L., Thingstad P., Taraldsen K., Lydersen S., Halsteinli V., Saltnes T., Lamb S.E. (2015). Comprehensive geriatric care for patients with hip fractures: A prospective, randomised, controlled trial. Lancet.

[18] Thingstad P., Taraldsen K., Saltvedt I., Sletvold O., Vereijken B., Lamb S.E., Helbostad J.L. (2016). The long-term effect of comprehensive geriatric care on gait after hip fracture: The Trondheim Hip Fracture Trial—A randomised controlled trial. Osteoporos. Int..

[19] Tarazona-Santabalbina F.J., Belenguer-Varea Á., Rovira E., Cuesta-Peredó D. (2016). Orthogeriatric care: Improving patient outcomes. Clin. Interv. Aging.

[20] Salpakoski A., Törmäkangas T., Edgren J., Sihvonen S., Pekkonen M., Heinonen A., Pesola M., Kallinen M., Rantanen T., Sipilä S. (2014). Walking recovery after a hip fracture: A prospective follow-up study among community-dwelling over 60-year old men and women. BioMed Res. Int..

[21] Kristensen M.T. (2011). Factors affecting functional prognosis of patients with hip fracture. Eur. J. Phys. Rehabil. Med..

[22] Martín-Martín L.M., Arroyo-Morales M., Sánchez-Cruz J.J., Valenza-Demet G., Valenza M.C., Jiménez-Moleón J.J. (2015). Factors influencing performance-oriented mobility after hip fracture. J. Aging Health.

[23] National Institute for Health and Care Excellence (NICE) Hip fracture: Management. NICE Guideline [NG124]. Updated January 2023. https://www.nice.org.uk/guidance/ng124.

[24] Sasabuchi Y., Matsui H., Lefor A.K., Fushimi K., Yasunaga H. (2018). Timing of surgery for hip fractures in the elderly: A retrospective cohort study. Injury.

[25] Ibe T., Ishizaki T., Oku H., Ota K., Takabatake Y., Iseda A., Ishikawa Y., Ueda A. (2008). Predictors of pressure ulcer and physical restraint prevalence in Japanese acute care units. Jpn. J. Nurs. Sci..

[26] Ariza-Vega P., Lozano-Lozano M., Olmedo-Requena R., Martín-Martín L., Jiménez-Moleón J.J. (2017). Influence of cognitive impairment on mobility recovery of patients with hip fracture. Am. J. Phys. Med. Rehabil..

[27] Jones C.A., Jhangri G.S., Feeny D.H., Beaupre L.A. (2017). Cognitive status at hospital admission: Postoperative trajectory of functional recovery for hip fracture. J. Gerontol. A Biol. Sci. Med. Sci..

[28] Uriz-Otano F., Uriz-Otano J.I., Malafarina V. (2015). Factors associated with short-term functional recovery in elderly people with a hip fracture. Influence of cognitive impairment. J. Am. Med. Dir. Assoc..

[29] Yoshikawa M., Goto E., Shin J.H., Imanaka Y. (2023). Regional disparities in Dementia-free Life Expectancy in Japan: An ecological study, using the Japanese long-term care insurance claims database. PLoS ONE.

[30] Tolea M.I., Morris J.C. (2016). Trajectory of mobility decline by type of dementia, Galvin JE. Alzheimer Dis. Assoc. Disord..

[31] Lynds M.E., Arnold C.M. (2023). Fall risk screening and assessment for people living with dementia: A scoping review. J. Appl. Gerontol..

[32] Rm A.M., Ekholm A., Zander V. (2021). Preventing falls and malnutrition among older adults in municipal residential care in Sweden: A registry study. Sage Open Nurs..

