# Supplementary material for: Functional Recovery Predictors in Hip Fractures: Insights from a Community Clinical Pathway
Source: J Clin Med. 2025 May 14;14(10):3430. doi: 10.3390/jcm14103430 (PMC12112208; doi:10.3390/jcm14103430)
Supplement: Supplementary file 1 [file jcm-14-03430-s001.zip › jcm-3563208-supplementary.pdf]

Table S1: Correlation coefficients between the factors.

Variables		
Number of patients		269
Median age (range)		85 (43–101)
Female, n (%)		197 (73.2%)
Fracture type, n (%)		
	Trochanteric fracture (AO/OTA 31-A)	142 (52.7%)
	Neck fracture (AO/OTA 31-B)	127 (47.3%)
Pre-injury mobility pattern, n (%)		
	Independent	182 (67.7%)
	Walking aid	74 (27.5%)
	Wheelchair	13 (4.8%)
Surgical method, n (%)		
	Osteosynthesis	94 (34.9)
	Bipolar head arthroplasty	175 (65.1)
Median pre-surgical days (range)		6 (0–49)
Median hospital days (range)		
	Acute care hospital	20 (4–77)
	Rehabilitation hospitals	52 (5–154)
	Total hospitals	73 (20–186)
Pre-injury dementia, n (%)		147 (54.6)
Nursing Needs Score, median (range)		
	Acute care hospital	6 (0–18)
	Rehabilitation hospitals	1 (0–19)
